# Validating the ChatGPT Usage Scale: psychometric properties and factor structures among postgraduate students

**DOI:** 10.1186/s40359-024-01983-4

**Published:** 2024-09-24

**Authors:** Mohamed Nemt-allah, Waleed Khalifa, Mahmoud Badawy, Yasser Elbably, Ashraf Ibrahim

**Affiliations:** Faculty of Education, Al-Azhar University, Dakahlia, Egypt

**Keywords:** ChatGPT, Artificial intelligence, Postgraduate education, Academic writing, Psychometrics, Factor analysis

## Abstract

**Background:**

The rapid adoption of ChatGPT in academic settings has raised concerns about its impact on learning, research, and academic integrity. This study aimed to develop and validate a comprehensive ChatGPT Usage Scale specifically tailored to postgraduate students, addressing the need for a psychometrically sound instrument to assess the multidimensional nature of ChatGPT usage in higher education.

**Methods:**

A cross-sectional survey design was employed, involving 443 postgraduate students from two Egyptian universities. The initial 39-item scale underwent Exploratory Factor Analysis (EFA) using principal component analysis with Varimax rotation. Confirmatory Factor Analysis (CFA) was conducted to assess the model fit and psychometric properties of the final 15-item measure. Internal consistency reliability was evaluated using Cronbach’s alpha and McDonald’s omega.

**Results:**

EFA revealed a three-factor structure explaining 49.186% of the total variance: Academic Writing Aid (20.438%), Academic Task Support (14.410%), and Reliance and Trust (14.338%). CFA confirmed the three-factor structure with acceptable fit indices (χ2(87) = 223.604, *p* < .001; CMIN/DF = 2.570; CFI = 0.917; TLI = 0.900; RMSEA = 0.060). All standardized factor loadings were statistically significant (*p* < .001), ranging from 0.434 to 0.728. The scale demonstrated good internal consistency (Cronbach’s α = 0.848, McDonald’s ω = 0.849) and composite reliability (CR = 0.855). The average variance extracted (AVE) was 0.664, supporting convergent validity.

**Conclusions:**

The validated ChatGPT Usage Scale provides a reliable and valid instrument for assessing postgraduate students’ engagement with ChatGPT across multiple dimensions. This tool offers valuable insights into AI-assisted academic practices, enabling more nuanced investigations into the effects of ChatGPT on postgraduate education.

## Introduction

The integration of artificial intelligence (AI) and natural language processing technologies into educational settings has sparked significant debate about their potential benefits and challenges [1, 2]. Among these emerging technologies, ChatGPT, an AI-powered conversational assistant developed by Anthropic, has gained particular prominence [3, 4]. Since its public release in late 2022, ChatGPT has quickly become popular among students and researchers for its ability to engage in human-like conversations, answer questions across a wide range of topics, and assist with various tasks including writing, programming, and problem-solving [5].

ChatGPT’s rapid adoption in academic settings, particularly among postgraduate students, raises concerns about its impact on learning, research, and academic integrity [6]. While some studies have highlighted ChatGPT’s potential for enhancing literature reviews, research assistance, and writing support, others have expressed concerns about over-reliance on AI-generated content and its implications for critical thinking and original scholarship [4, 7]. This tension underscores the need for a comprehensive understanding of how postgraduate students are using ChatGPT, their perceptions of its utility, and the ethical considerations surrounding its use in academic work [8].

Previous research has explored various aspects of AI assistants in education, including adoption factors [9], effects on higher education [10], and strategies for course integration [11]. However, there is a notable gap in the literature regarding validated instruments that can assess the multidimensional nature of ChatGPT usage, particularly among postgraduate students. This gap limits our ability to quantitatively measure and analyze the impact of ChatGPT on postgraduate education, hindering evidence-based policy development and curriculum design.

The approach is based on various theoretical frameworks that provide diverse perspectives on technology adoption and usage in educational settings. The Technology Acceptance Model (TAM) provides insights into the factors influencing students’ acceptance of new technologies [12, 13]. Self-Determination Theory (SDT) helps us understand the intrinsic and extrinsic motivations driving ChatGPT use [14, 15]. Additionally, Cognitive Load Theory (CLT) offers a lens through which to examine the potential cognitive benefits and challenges associated with AI assistance in academic tasks [16, 17].

Previous studies have tested the reliability and validity of the Technology Acceptance Model Edited to Assess ChatGPT Adoption (TAME-ChatGPT) survey, which measures perceived risks, attitudes, anxiety, usefulness, ease of use, and behavioral factors among university students [18]. Abdaljaleel et al. reestablished the instrument’s structural validity with Arab students, emphasizing perceived ease of use, usefulness, positive attitudes, social influence, behavioral and cognitive factors, low computer anxiety, and perceived risks [19].

The present study aims to address this gap by developing and validating a comprehensive ChatGPT Usage Scale specifically tailored to postgraduate students. This scale is designed to capture multiple dimensions of ChatGPT usage, including its role in academic writing, support for various academic tasks, and students’ reliance on and trust in the technology. By providing a psychometrically sound instrument, this study seeks to enable more nuanced and rigorous investigations into the effects of ChatGPT on postgraduate education.

As AI technologies continue to evolve and become more integrated into higher education, it is crucial to have robust, empirically-validated measures to assess their impact. The ChatGPT Usage Scale developed in this study represents an important step towards this goal, offering a foundation for future research and contributing to the ongoing dialogue about the role of AI in academia. By providing a nuanced understanding of how postgraduate students engage with ChatGPT, this research aims to inform evidence-based strategies for harnessing the potential benefits of AI assistants while addressing potential challenges and ethical concerns in postgraduate education.

## Method

### Research design

This study employed a cross-sectional survey design to validate the ChatGPT Usage Scale among postgraduate students in Egyptian universities.

### Participants

The study participants were 443 postgraduate students enrolled in Egyptian universities, selected through stratified random sampling. The sample was drawn from two institutions: Faculty of Education at Kafr el-Sheikh University (population *N* = 856) and Faculty of Education at Al-Azhar University (population *N* = 1113). Specifically, 124 participants were from Kafr el-Sheikh University, and 319 were from Al-Azhar University. The sample size of 443 represents approximately 22.5% of the combined postgraduate student population from both universities (*N* = 1969), exceeding the commonly recommended minimum of 10 participants per item for scale validation studies [20].

The sample comprised 194 (43.8%) males and 249 (56.2%) females, with an average age of 27.4 years (*SD* = 4.8, range = 21–48 years). Participants were distributed across different postgraduate levels: 286 postgraduate diploma students (64.6%), 155 master’s students (35.0%), and 102 doctoral students (23.0%).

Eligibility criteria included being a current postgraduate student at one of the participating universities and the ability to read and comprehend the scale items in English.

### Measures

The primary instrument used was *the ChatGPT Usage Scale*, developed specifically for this study. The initial ChatGPT Usage Scale consisted of 39 items designed to assess postgraduate students’ usage patterns, perceptions, and experiences with ChatGPT across three key dimensions: Academic Writing Aid, Academic Task Support, and Reliance and Trust. All items were rated on a 5-point Likert scale ranging from 1 (strongly disagree) to 5 (strongly agree). One dimension concerning ethics and academic integrity was reverse-coded.

### Procedure

Participants were recruited through the sharing of emails and posts on social media platforms with directives from different higher learning institutions in Egypt. For those who were interested in participating, a link to the Google Forms survey was made available. Following the signing of consent forms for the study, the participants answered the ChatGPT Usage Scale and some basic demographic questions. To ensure that participants responded adequately to the items that needed elaboration, the survey was run for three weeks in February 2023.

### Data analysis

Data analysis was conducted using IBM SPSS Statistics 23 and AMOS 23. The analysis process involved two main steps:

- Exploratory Factor Analysis (EFA): An initial EFA was performed to assess the factorial validity of the 39-item scale and identify poor or cross-loading items. Principal component analysis with Varimax rotation was used.
- Confirmatory Factor Analysis (CFA): Following the EFA, a CFA was conducted to assess the model fit and psychometric properties of the final 15-item measure. Goodness-of-fit indices including chi-square, CFI, GFI, TLI, RMSEA, and SRMR were examined. Acceptable model fit criteria were set as CFI, GFI, and TLI ≥ 0.90, and RMSEA ≤ 0.08 [21, 22].

Internal consistency reliability was assessed using Cronbach’s alpha and McDonald’s omega, with values ≥ 0.7 considered acceptable [23]. Item-total correlations were also examined to assess individual item performance.

## Results

An initial Exploratory Factor Analysis (EFA) was conducted on the original 39-item ChatGPT Usage Scale. Based on the results of this analysis, the scale was refined to a final 15-item version. This 15-item scale was then subjected to further analysis. Table 1 presents the factor loadings, means, and standard deviations for all 15 items of the scale, as well as item total correlation.

**Table 1:** Factor loadings of 15-item ChatGPT scale

Item	Academic Writing Aid	Academic Task Support	Reliance and Trust	M	SD	Item total correlation
34. I use ChatGPT to paraphrase complex academic concepts for better understanding.	0.748			3.67	1.112	0.598
38. ChatGPT helps me develop counterarguments to strengthen my academic writing.	0.743			3.48	1.148	0.610
13. I use ChatGPT to generate ideas for my academic writing.	0.675			2.80	1.285	0.680
5. I use ChatGPT to generate drafts that I edit and finalize myself.	0.613			2.90	1.166	0.631
18. ChatGPT assists me in finding relevant sources or references for my research.	0.548			2.95	1.132	0.613
23. ChatGPT saves me time and effort in academic writing.	0.536			3.54	1.142	0.653
8. ChatGPT allows me to explore ideas that I may not have considered on my own	0.502			3.54	1.163	0.513
30. I use ChatGPT to overcome writer’s block when starting essays.		0.751		3.24	1.185	0.578
14. ChatGPT assists me in organizing my thoughts and creating outlines.		0.649		3.69	1.194	0.541
3. I use ChatGPT to quickly find relevant information for research tasks.		0.630		2.76	1.193	0.483
10. ChatGPT helps me create study materials like flashcards or summaries more efficiently.		0.548		2.62	1.194	0.493
12. ChatGPT provides a starting point but cannot replace my own thinking and writing.			0.733	2.78	1.107	0.608
2. I trust the quality and accuracy of ChatGPT outputs.			0.698	3.07	1.155	0.386
16. I use ChatGPT to get feedback and suggestions for improving my writing.			0.632	3.03	1.148	0.599
22. I use ChatGPT as a brainstorming partner rather than a source of final answers.			0.570	3.05	1.182	0.493

The EFA, conducted using principal component analysis with Varimax rotation, revealed a three-factor structure explaining 49.186% of the total variance. The first factor, Academic Writing Aid, accounted for 20.438% of the variance. The second factor, Academic Task Support, accounted for 14.410%, and the third factor, Reliance and Trust, accounted for 14.338% after rotation.

The item-total correlations were examined to assess how well each item correlated with the overall scale. For the items where this information was available, the item-total correlations ranged from 0.386 to 0.680, indicating moderate to strong relationships between individual items and the overall scale. The highest item-total correlation was observed for item 13 (“I use ChatGPT to generate ideas for my academic writing”) at 0.680, while the lowest was for item 8 (“I trust the quality and accuracy of ChatGPT outputs”) at 0.386. These values suggest that the items are generally consistent with the overall construct being measured by the scale.

**Fig. 1:**
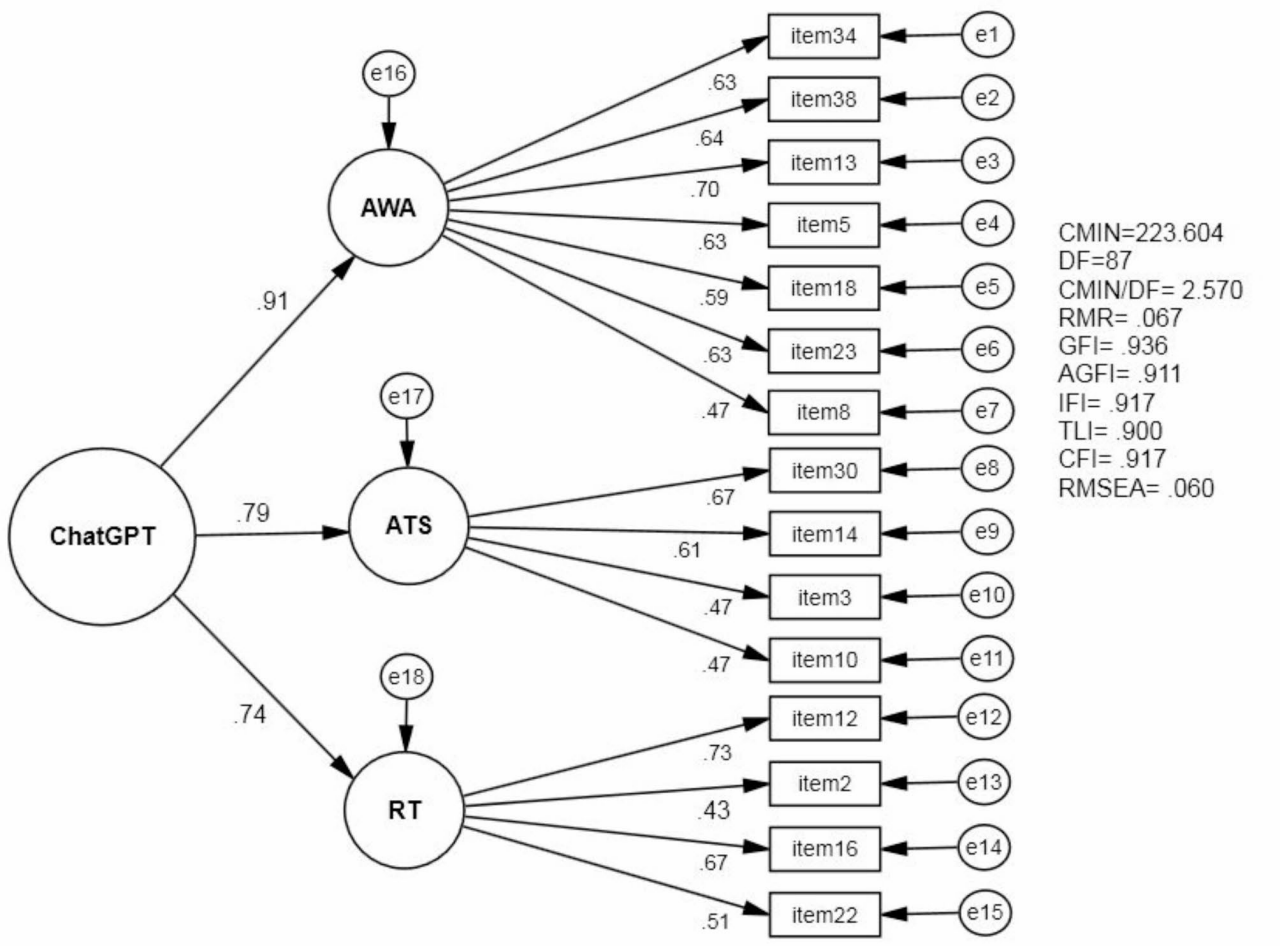
Standardized CFA for the three-factor 15-item structure model

To validate the three-factor structure identified in the EFA, a confirmatory factor analysis (CFA) was conducted. The model specified the three factors (AWA, ATS, and RT) as latent variables, with their respective items as observed variables. A second-order factor was also included to represent the overall ChatGPT usage construct (Fig. 1).

The CFA results indicated an acceptable fit for the proposed model: χ2(87) = 223.604, *p* < .001; CMIN/DF = 2.570; CFI = 0.917; TLI = 0.900; RMSEA = 0.060 (90% CI: 0.050–0.069). All standardized factor loadings were statistically significant (*p* < .001) and ranged from 0.434 to 0.728, exceeding the minimum threshold of 0.4 [24]. The second-order factor loadings were also strong, with standardized coefficients of 0.909 for AWA, 0.787 for ATS, and 0.739 for RT, indicating that these first-order factors were well-represented by the higher-order ChatGPT usage construct.

The composite reliability (CR) for the overall scale was 0.855, indicating good internal consistency reliability. The average variance extracted (AVE) was 0.664, supporting the convergent validity of the scale [25]. Internal consistency was also assessed using McDonald’s omega (ω) and Cronbach’s alpha (α) coefficients. For the overall scale, ω = 0.849 (95% CI: 0.829–0.870) and α = 0.848 (95% CI: 0.826–0.867), indicating good internal consistency.

In summary, the results of the EFA and CFA support a three-factor structure for the ChatGPT Usage Scale, comprising Academic Writing Aid, Academic Task Support, and Reliance and Trust dimensions. The scale demonstrated good psychometric properties, including acceptable model fit, strong factor loadings, and adequate reliability.

## Discussion

The present study aimed to develop and validate a scale to measure postgraduate students’ usage patterns, perceptions, and experiences with ChatGPT. The results support a three-factor structure for the ChatGPT Usage Scale, comprising Academic Writing Aid, Academic Task Support, and Reliance and Trust dimensions. This multidimensional scale demonstrates good psychometric properties, including acceptable model fit, strong factor loadings, and adequate reliability, providing a valuable tool for assessing ChatGPT use among postgraduate students.

The emergence of the Academic Writing Aid factor aligns with previous research highlighting ChatGPT’s potential as a writing assistant in academic contexts [4, 7]. This factor encompasses various aspects of writing support, including idea generation, drafting, paraphrasing, and developing counterarguments. The high factor loadings for items related to paraphrasing complex concepts (0.748) and developing counterarguments (0.743) suggest that postgraduate students find ChatGPT particularly useful for enhancing their academic writing skills. This finding is consistent with the growing body of literature on AI-assisted writing in higher education [1, 2] and underscores the potential of AI tools to support students in developing more sophisticated academic arguments.

The Academic Task Support factor reflects the broader utility of ChatGPT in various academic tasks beyond writing. This factor includes items related to overcoming writer’s block, organizing thoughts, creating study materials, and finding relevant information for research tasks. The emergence of this factor supports the notion that AI assistants like ChatGPT can serve as versatile tools in postgraduate education, potentially enhancing students’ efficiency and productivity across various academic activities [3, 11]. However, the relatively lower factor loadings for some items in this dimension (e.g., 0.548 for creating study materials) suggest that students may perceive ChatGPT as more helpful for certain tasks than others, warranting further investigation into the specific areas where AI assistance is most beneficial.

The Reliance and Trust factor provides insights into students’ attitudes towards ChatGPT and their level of dependence on the tool. This factor includes items assessing trust in ChatGPT’s outputs, use of the tool for feedback, and recognition of its limitations. The emergence of this factor aligns with previous research on technology acceptance and trust in AI systems [18, 19]. The moderate factor loadings for items in this dimension (ranging from 0.570 to 0.733) suggest that postgraduate students have a nuanced view of ChatGPT, recognizing both its potential benefits and limitations. This finding is particularly important in the context of ongoing debates about the ethical implications and potential overreliance on AI tools in academic settings [2, 26].

The overall good fit of the three-factor model, as evidenced by the CFA results (CFI = 0.917; TLI = 0.900; RMSEA = 0.060), supports the construct validity of the ChatGPT Usage Scale. The strong second-order factor loadings (0.909 for Academic Writing Aid, 0.787 for Academic Task Support, and 0.739 for Reliance and Trust) indicate that these dimensions are well-represented by the higher-order ChatGPT usage construct. This hierarchical structure provides a comprehensive framework for understanding and measuring ChatGPT use among postgraduate students, capturing both specific use cases and broader attitudinal factors.

The scale’s good internal consistency reliability, as demonstrated by the composite reliability (CR = 0.855) and McDonald’s omega (ω = 0.849), suggests that the items within each factor are measuring similar constructs. This reliability, coupled with the scale’s content validity established through the development process and its factorial validity confirmed through EFA and CFA, positions the ChatGPT Usage Scale as a robust instrument for future research in this area.

Comparing our findings with existing literature, the multidimensional nature of the ChatGPT Usage Scale aligns with previous attempts to measure AI technology acceptance in educational contexts [18, 19]. However, our scale offers a more focused assessment of ChatGPT usage specifically among postgraduate students, providing a nuanced understanding of how this population engages with the tool for academic purposes. The emergence of distinct factors for writing aid and broader academic task support extends beyond general technology acceptance models, offering insights into the specific ways in which postgraduate students utilize ChatGPT in their academic work.

Despite these strengths, several limitations of the study should be acknowledged. First, the sample was limited to postgraduate students in Egyptian universities, potentially limiting the generalizability of the findings to other cultural or educational contexts. Future research should validate the scale across diverse populations and educational settings to establish its cross-cultural validity. Second, the self-report nature of the scale may be subject to social desirability bias, particularly given the ongoing debates surrounding the ethical use of AI tools in academic settings. Future studies could incorporate behavioral measures or objective usage data to complement self-reported measures.

Additionally, the rapidly evolving nature of AI technology presents a challenge for any measurement tool in this domain. As ChatGPT and similar AI assistants continue to develop and new features are introduced, the scale may need to be updated to reflect these changes. Regular validation and potential revision of the scale will be necessary to ensure its continued relevance and accuracy in measuring ChatGPT usage.

## Conclusion

The ChatGPT Usage Scale is a valuable tool for understanding the impact of AI assistants on academic practices in higher education. It measures postgraduate students’ engagement with ChatGPT across multiple dimensions, including writing support, broader academic tasks, and issues of reliance and trust. Future research should explore the relationship between ChatGPT usage patterns and academic outcomes, investigate moderating factors, and examine the long-term implications of AI assistant use on skill development and academic integrity. Qualitative studies could provide deeper insights into students’ decision-making processes. Tools like the ChatGPT Usage Scale are essential for evidence-based decision-making and policy development in the rapidly evolving field of AI.

## Data Availability

The datasets generated and analyzed during the current study are available from the corresponding author, Mohamed Nemt-allah, upon reasonable request. Requests for data access can be made by contacting mohamednamatallah.2026@azhar.edu.eg.
